# Outbreak of Locally Acquired Mosquito-Transmitted (Autochthonous) Malaria — Florida and Texas, May–July 2023

**DOI:** 10.15585/mmwr.mm7236a1

**Published:** 2023-09-08

**Authors:** Dawn Blackburn, Michael Drennon, Kelly Broussard, Andrea M. Morrison, Danielle Stanek, Elizabeth Sarney, Christina Ferracci, Steve Huard, Wade Brennan, John Eaton, Sara Nealeigh, Natalie Barber, Rebecca A. Zimler, Jeremy N. Adams, Carina Blackmore, Manuel Gordillo, Robert Mercado, Harold Vore, Kelly Scanlan, Ian Motie, Leslie Stanfield, Ahmed Farooq, Kimberly Widel, Kelly Tomson, Nancy Kerr, John Nasir, Marshall Cone, Connor Rice, Thomas Larkin, Edwin Hernandez, Jennifer Bencie, Christopher R. Lesser, Max Dersch, Samantha Ramirez-Lachmann, Marah Clark, Susan Rollo, Amira Bashadi, Ronald Tyler, Bethany Bolling, Brent Moore, Brendan Sullivan, Eric Fonken, Raquel Castillo, Yaziri Gonzalez, Gustavo Olivares, Kimberly E. Mace, Dean Sayre, Audrey Lenhart, Alice Sutcliffe, Ellen Dotson, Claudia Corredor, Emma Rogers, Brian H. Raphael, Sarah G. H. Sapp, Yvonne Qvarnstrom, Alison D. Ridpath, Peter D. McElroy

**Affiliations:** ^1^Epidemic Intelligence Service, CDC; ^2^Division of Parasitic Diseases and Malaria, National Center for Emerging and Zoonotic Infectious Diseases, CDC; ^3^Florida Department of Health in Sarasota County, Sarasota, Florida; ^4^Texas Department of State Health Services Zoonosis Control Branch; ^5^Division of Disease Control and Health Protection, Florida Department of Health; ^6^Sarasota County Mosquito Management, Sarasota, Florida; ^7^Sarasota Memorial Hospital, Sarasota, Florida; ^8^Doctors Hospital, Sarasota, Florida; ^9^Bureau of Public Health Laboratories, Florida Department of Health, Jacksonville, Florida; ^10^Bureau of Public Health Laboratories, Florida Department of Health, Tampa, Florida; ^11^Florida Department of Health in Manatee County, Bradenton, Florida; ^12^Manatee County Mosquito Control District, Palmetto, Florida; ^13^Bureau of Scientific Evaluation and Technical Assistance, Florida Department of Agriculture and Consumer Services; ^14^Texas Department of State Health Services Region 11, Harlingen, Texas; ^15^Texas Department of State Health Services Laboratory Services Section; ^16^Texas Department of State Health Services Region 4/5 North, Tyler, Texas; ^17^Texas Department of State Health Services Region 6/5 South, Houston, Texas; ^18^Cameron County Public Health, San Benito, Texas; ^19^Brownsville Public Health Department, Brownsville, Texas.

**Summary**

**What is already known about this topic?**

Locally acquired mosquito-transmitted (autochthonous) *Plasmodium vivax* malaria was most recently reported in the United States in 2003.

**What is added by this report?**

Eight cases of autochthonous malaria were reported to CDC by state health departments in Florida (seven) and Texas (one) during May 18–July 17, 2023. Case surveillance, mosquito surveillance and control activities, and public outreach are ongoing.

**What are the implications for public health practice?**

The risk for autochthonous malaria in the United States remains very low. Prompt diagnosis and treatment of persons with malaria and reporting of cases to health departments and CDC is important to ensuring favorable clinical outcomes and a timely public health response. Malaria and other mosquitoborne diseases can be prevented by preventing mosquito bites.

## Abstract

Eight cases of locally acquired, mosquito-transmitted (i.e., autochthonous) *Plasmodium vivax* malaria, which has not been reported in the United States since 2003, were reported to CDC from state health departments in Florida and Texas during May 18–July 17, 2023. As of August 4, 2023, case surveillance, mosquito surveillance and control activities, and public outreach and education activities continue in both states. U.S. clinicians need to consider a malaria diagnosis in patients with unexplained fever, especially in areas where autochthonous malaria has been recently reported, although the risk for autochthonous malaria in the United States remains very low. Prompt diagnosis and treatment of malaria can prevent severe disease or death and limit ongoing transmission to local *Anopheles* mosquitoes and other persons. Preventing mosquito bites and controlling mosquitoes at home can prevent mosquitoborne diseases, including malaria. Before traveling internationally to areas with endemic malaria, travelers should consult with a health care provider regarding recommended malaria prevention measures, including potentially taking malaria prophylaxis. Malaria is a nationally notifiable disease; continued reporting of malaria cases to jurisdictional health departments and CDC will also help ensure robust surveillance to detect and prevent autochthonous malaria in the United States.

## Investigation and Results

**FIGURE 1:**
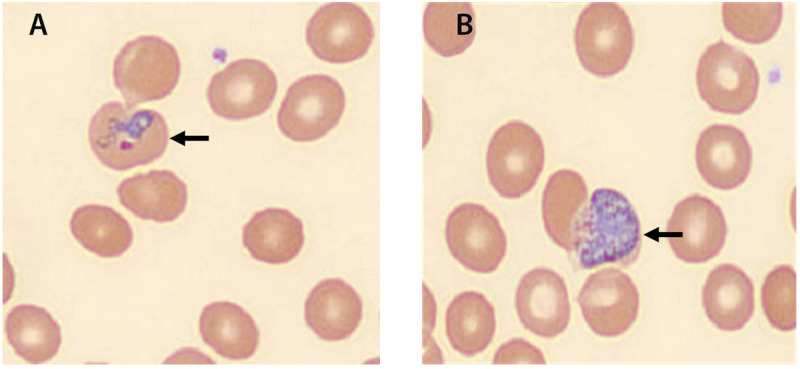
Thin blood smear from patient showing *Plasmodium vivax *ring-form trophozoite (A) and gametocyte (B) — Florida, May 2023

On May 18, 2023, the Florida Department of Health (FDOH) requested telediagnosis assistance from DPDx, CDC’s interactive parasitic diseases website (https://www.cdc.gov/dpdx/index.html), to confirm *Plasmodium* species in a patient with suspected malaria who had no known risk factors (i.e., history of international travel, intravenous drug use, blood transfusion, or organ transplantation). CDC confirmed *Plasmodium vivax* (Figure 1), triggering an investigation into the first mosquito-transmitted (i.e., autochthonous) malaria case in the United States since 2003, when eight cases of autochthonous *P. vivax* malaria were identified in Palm Beach County, Florida (*1*).

On June 7, 2023, a hospital in Texas requested CDC DPDx telediagnosis assistance to confirm malaria in a patient who also did not report any known risk factors. CDC similarly confirmed *P. vivax* and notified the Texas Department of State Health Services (TDSHS). The most recent documented autochthonous malaria cases in Texas occurred in 1994 (*2*).

During June 19–July 17, 2023, six additional cases of autochthonous *P. vivax* malaria were reported to CDC by FDOH. None of the eight patients had received a previous malaria diagnosis. CDC is supporting both state health departments in the ongoing investigations of and response to these cases.^†^ This activity was reviewed by CDC and was conducted consistent with applicable federal law and CDC policy.^§^

**FIGURE 2:**
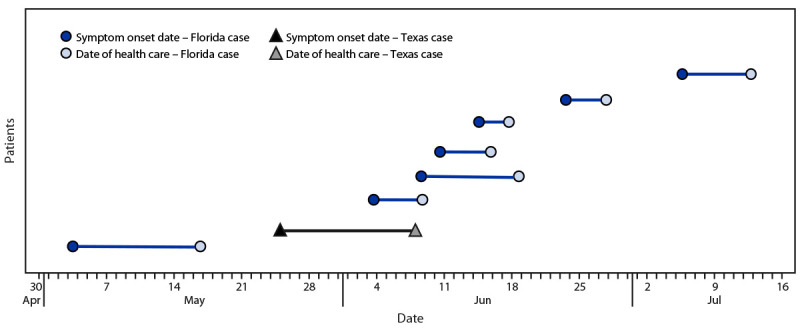
Intervals between symptom onset and health care date resulting in malaria diagnosis among patients with autochthonous malaria (N = 8) — Florida and Texas, May–July 2023

All seven reported Florida cases occurred in persons who lived within a 4-mile (6.4-km) radius in Sarasota County. An imported case of *P. vivax* malaria (symptom onset of April 20) was previously reported in the same immediate area. In Texas, an imported case of *P. vivax* malaria (symptom onset of May 2) was previously reported in the same area in Cameron County where the patient with autochthonous infection was likely exposed. Whether the imported cases in Florida and Texas are related to these autochthonous malaria cases is currently not known. No evidence links the outbreaks in the two states (Figure 2).

Patients with autochthonous malaria sought health care for a range of clinical signs and symptoms, and cases were diagnosed using a combination of rapid diagnostic tests (RDTs), blood smears, or polymerase chain reaction (PCR) testing (Table). *P. vivax* was identified on blood smears of all eight patients. All patients reported fever, six reported chills, five reported abdominal pain, and five reported vomiting. Thrombocytopenia was reported for all eight patients, and six were anemic. Seven of the eight patients were hospitalized, none developed severe malaria,^¶^ and no deaths were reported. Seven of the eight patients did not report any of the known risk factors for malaria. One patient reported intravenous drug use 1 week before symptom onset but did not report unsafe injection practices; drug use was not determined to be a risk factor for this patient’s malaria diagnosis. Three of the patients in Florida reported experiencing homelessness.

**TABLE:** Epidemiologic and clinical characteristics of locally acquired mosquito-transmitted malaria cases (N = 8) — Florida and Texas, May–July 2023

Characteristic	No. of cases (%)
**Age group, yrs**	
20–39	4 (50)
40–59	3 (38)
60–79	1 (13)
**Sex**	
Men	6 (75)
Women	2 (25)
**Signs and symptoms at time of seeking medical attention**	
Fever	8 (100)
Chills	6 (75)
Abdominal pain	5 (63)
Vomiting	5 (63)
Nausea	4 (50)
Sweats	3 (38)
Diarrhea	3 (38)
Fatigue or weakness	3 (38)
Anorexia	2 (25)
Myalgia	2 (25)
Body ache	1 (13)
Headache	1 (13)
Back pain	1 (13)
Cough	1 (13)
Melena	1 (13)
Shortness of breath	1 (13)
**Diagnostic test**	
Positive rapid diagnostic test result	5 (63)
Positive blood smear test result	8 (100)
Positive PCR test result*	8 (100)

Abbreviation: PCR = polymerase chain reaction.

* *Plasmodium vivax* was confirmed by CDC through PCR targeting 18S rRNA.

Patients with confirmed *P. vivax* malaria require immediate treatment for the acute blood-stage infection followed by antirelapse treatment of liver-stage infection to prevent recurring parasitemia months or years later. As of August 4, 2023, five patients had been treated with artemether-lumefantrine and three with atovaquone-proguanil. All patients were prescribed primaquine for antirelapse treatment. Because primaquine cannot be used in patients with glucose-6-phosphate dehydrogenase (G6PD) deficiency, quantitative G6PD activity assays confirmed that all patients had normal enzyme activity. All patients have recovered.

## Florida Public Health Response

### Active Case Surveillance

On May 17, 2023, enhanced case finding through syndromic surveillance was initiated, including a retrospective review of data during the preceding 4 weeks. On May 26, Florida began active surveillance for additional cases, to be continued until ≥8 weeks pass without identification of additional cases. Local hospitals in Sarasota and Manatee counties were requested to begin performing malaria tests for persons meeting specific clinical criteria.** All seven cases were reported by the providers to the county health department; providers were the first reporting source for six of the cases, and one case was detected first through syndromic surveillance. CDC is providing ongoing laboratory support by performing PCR confirmatory testing; genotyping of parasite DNA from each case is underway. As of August 4, a total of 30 patients identified through syndromic surveillance received negative malaria PCR test results.^††^

### Mosquito Surveillance and Control Activities

After immediate notification of the first suspected case to mosquito control programs in Sarasota and neighboring Manatee County, enhanced mosquito surveillance commenced in the affected area, including use of CDC light traps and CDC UpDraft Blacklight (ultraviolet) traps in known *Anopheles* breeding areas within 0.9 miles (1.5 km) of the first identified case. Trapped mosquitoes morphologically identified as *Anopheles* were assayed for evidence of *Plasmodium* infection by CDC’s Division of Parasitic Diseases and Malaria Entomology Branch.^§§^ As of August 4, a total of 407 *Anopheles* mosquitoes have been tested. No sporozoite-positive mosquitoes have been detected, but three *Anopheles crucians* abdomens were positive for *P. vivax* DNA, suggesting that these mosquitoes had recently fed on a *P. vivax–*infected person.^¶¶^

On May 24, 2023, Florida began enhanced mosquito control activities in the affected area, including aerial and ground spraying for adult mosquitoes. Because the area of public health concern crossed county boundaries, interagency communication and coordination were increased, including aerial larvicide application to treat large areas of wetland habitat not easily accessible by other means. Products containing spinosad (a natural substance toxic to some insects), monomolecular films (which reduce the surface tension of water preventing mosquito larvae from attaching to the surface), methoprene (an insect growth regulator), and *Bacillus thuringiensis israelensis* (*Bti*) dunks (a common larvicide), were used to treat standing water for mosquito larvae.

### Public Outreach

After confirmation of the first case, FDOH in Sarasota and Manatee County issued a mosquitoborne illness advisory on May 26. On June 19, after confirmation of the second autochthonous case, both county health departments upgraded the advisory to a mosquitoborne illness alert. On June 26, FDOH issued a statewide mosquitoborne illness advisory and a health care provider notification after confirmation of the third and fourth cases.

FDOH collaborated with organizations serving unhoused persons in the area and provided insect repellent, bed nets, and education on mosquito bite prevention. FDOH recommended that any persons experiencing homelessness seek care if they developed symptoms. FDOH also repeatedly distributed information on the diagnosis and treatment of malaria to clinicians in the area.

## Texas Public Health Response

### Active Case Surveillance

In May, four persons who worked outdoors at the same time and location as the first identified Texas patient self-reported an illness consistent with malaria to their supervisors. TDSHS successfully contacted three of the four persons to ascertain clinical details and recommend malaria testing; one person was lost to follow-up. One person received a negative whole blood PCR malaria test result at the TDSHS laboratory. One of the contacted persons lives outside of Texas; TDSHS coordinated with the state health department in that person’s state of residence to recommend malaria testing, but the individual chose not to pursue malaria testing. The third contacted person also chose not to pursue malaria testing.

### Mosquito Surveillance and Control Activities

On June 13, 2023, mosquito control agencies in the area began enhanced mosquito surveillance and control activities at three target sites in Cameron County: 1) the worksite where the patient with an autochthonous case spent time outside at night, 2) his temporary residence, and 3) the temporary residence of the patient with imported malaria. Mosquito surveillance and control activities were also conducted around the patient’s residence (outside of Cameron County). Mosquitoes were collected using CDC light traps; those morphologically identified as *Anopheles* were assayed for evidence of *Plasmodium* infection by CDC’s Division of Parasitic Diseases and Malaria Entomology Branch. As of August 4, a total of 71 *Anopheles* mosquitoes collected from the target sites and the patient’s residence were sent to CDC for *Plasmodium* testing; all test results were negative.

On June 16, five adulticidal treatments were conducted with an ultralow volume fogger. *Bti* dunks were used to treat standing water for mosquito larvae. Adulticidal treatments were also conducted in May in response to high mosquito activity near the work site of the patient with autochthonous malaria.

### Public Outreach

On June 23, TDSHS issued a statewide health advisory regarding an autochthonous malaria case in Texas. Cameron County Public Health issued a similar public health advisory the same day. Information on malaria was also shared with Cameron County infection control practitioners and emergency response partners. In addition, Cameron County Public Health initiated education and outreach measures with local health care providers and residents; the CDC Health Alert Network (HAN) Health Advisory (*3*) was forwarded to other local health departments and health care providers in the region.

## CDC Response

On June 26, 2023, CDC issued a HAN Health Advisory to notify clinicians, public health authorities, and the public about the autochthonous malaria cases in Florida and Texas (*3*). On July 20, CDC convened a Clinician Outreach and Communication Activity webinar to provide information to clinicians on the diagnosis and treatment of malaria. CDC continues to provide technical assistance in laboratory diagnostics, treatment options, active case detection strategies, epidemiology, and entomology and continues to ensure timely communication with other state and federal agencies regarding response activities.

## Discussion

Malaria was eliminated as a public health threat in the United States in the mid-1950s, and the World Health Organization certified the United States malaria-free in 1970. Currently, approximately 2,000 cases of malaria are diagnosed in the United States annually, although most cases are imported from countries where malaria remains endemic (*4*,*5*).

As of August 4, no additional autochthonous *P. vivax* cases have been detected in Florida or Texas since early July, and there has been no evidence of infected *Anopheles* mosquitoes since early June.*** The autochthonous malaria cases during this investigation highlight the importance of controlling cases globally to prevent future autochthonous cases in the United States. Species of *Anopheles* mosquitoes that are biologically capable of transmitting malaria are found throughout many regions of the United States.^†††^

Although the risk for autochthonous malaria in the United States remains very low,^§§§^ U.S. clinicians need to consider a malaria diagnosis in patients with an unexplained fever, especially in areas where autochthonous malaria has been recently reported. The occurrence of the current autochthonous cases underscores the potential for imported malaria cases in areas with competent vectors to produce local mosquito transmission of malaria parasites. Targeted mosquito surveillance based on the location of human cases is important in predicting human risk and preventing disease in humans. Prompt diagnosis and treatment^¶¶¶^ of persons with malaria can prevent severe disease or death and limit ongoing transmission to local *Anopheles* mosquitoes and other persons. Continued reporting of malaria cases to jurisdictional health departments and CDC will help ensure robust surveillance to prevent, detect, and respond to autochthonous malaria in the United States. Before traveling internationally to areas where malaria is endemic, travelers should consult with their health care provider regarding recommended malaria prevention measures, including potentially taking malaria prophylaxis. In addition, preventing mosquito bites and controlling mosquitoes at home can prevent malaria, and other mosquitoborne diseases.****

## Footnotes

† This outbreak report does not include the recent locally acquired case of *P. falciparum* malaria that was diagnosed in the National Capital Region, Maryland, in August 2023.

§ 45 C.F.R. part 46, 21 C.F.R. part 56; 42 U.S.C. Sect. 241(d); 5 U.S.C. Sect. 552a; 44 U.S.C. Sect. 3501 et seq.

¶
https://www.cdc.gov/malaria/about/disease.html

** The original syndromic surveillance query included review of hospital and urgent care chief complaint and discharge diagnoses mentioning 1) malaria, 2) homelessness with fever or chills, and 3) subjective or objective fever or chills with thrombocytopenia or anemia, and medical records and malaria PCR testing were requested as indicated. Independent additional malaria testing at hospitals also occurred on the basis of these criteria. The original syndromic surveillance query was updated on the basis of syndromic surveillance data for the first four cases, adding 1) splenomegaly and 2) abdominal pain with one of the original clinical criteria search terms.

†† BinaxNOW Malaria RDTs were also offered to the hospitals as a malaria screening tool. Because of operational challenges, there was limited use of RDTs.

§§ Specific tests include screening for *Plasmodium* circumsporozoite proteins in heads and thoraces (referred to as “sporozoite-positive”) via bead immunoassay and DNA extractions from abdomens to detect *Plasmodium* cytochrome c oxidase subunit I via PCR.

¶¶ These three mosquitoes were collected from two trapping locations in close proximity during three separate collection nights in early June. The presence of DNA in the mosquito abdomens suggested that these mosquitoes had recently fed on a person infected with *P. vivax*, but the lack of parasites in their heads and thoraces meant they were not considered infectious to humans.

*** The heightened period of public health vigilance will extend at least through mid-September 2023 as long as no additional human cases or infected *Anopheles* vectors are detected. At that point, this outbreak of autochthonous malaria would be considered over. This takes into consideration the incubation period of *P. vivax* in humans, duration of parasite development in the mosquito preceding transmission potential to humans, mosquito survival probabilities, and potential delays in health care–seeking behavior among persons with new cases.

††† The risk might be higher in areas where local climatic conditions allow *Anopheles* populations to survive during most of the year and where travelers from areas with endemic malaria are found.

§§§ Clinicians practicing in areas of the United States where autochthonous malaria cases have occurred need to follow guidance from their state and local health departments.

¶¶¶
https://www.cdc.gov/malaria/diagnosis_treatment/index.html
